# Pediculus Humanus Capitis in an Immuno Compromised Patient

**Published:** 1989-02

**Authors:** P. A. Bloom, M. Houghton

**Affiliations:** Bristol Eye Hospital Lower Maudlin Street Bristol BS1 2LX; Bristol Eye Hospital Lower Maudlin Street Bristol BS1 2LX


### Letter to the Editor

### PEDICULUS HUMANUS CAPITIS IN AN IMMUNO COMPROMISED PATIENT

Sir,

A 58 year old welder was treated for oat cell carcinoma of the bronchus on a general medical ward, with a combination of Adriamycin, Cyclophosphamide and Etoposide. Within two weeks he had developed drug induced bone marrow suppression and subtotal alopecia, sparing only his eyelashes, eyebrows and nipple hair. He subsequently contracted pediculosis in these areas, which could not be isolated from his family, visitors, ward staff or other patients.

We postulate that this was a case of opportunistic pediculosis in an immuno compromised patient and it is interesting that it occurred in subtotal alopecia in the patient's little remaining hair; it is the first reported case of its kind and stresses the importance of isolation. We would be interested to hear of any similar cases

P. A. Bloom
M. Houghton

Bristol Eye Hospital
Lower Maudlin Street
Bristol BS1 2LX

